# Supplementary material for: Safeguarding climate-resilient mangroves requires only a moderate increase in the global protected area
Source: Nat Commun. 2026 Jan 27;17:2063. doi: 10.1038/s41467-026-68877-4 (PMC12949150; doi:10.1038/s41467-026-68877-4)
Supplement: Supplementary file 1 — Supplementary Information [file 41467_2026_68877_MOESM1_ESM.pdf]

Safeguarding climate-resilient mangroves requires only a moderate increase in the global protected area

Supplementary Material

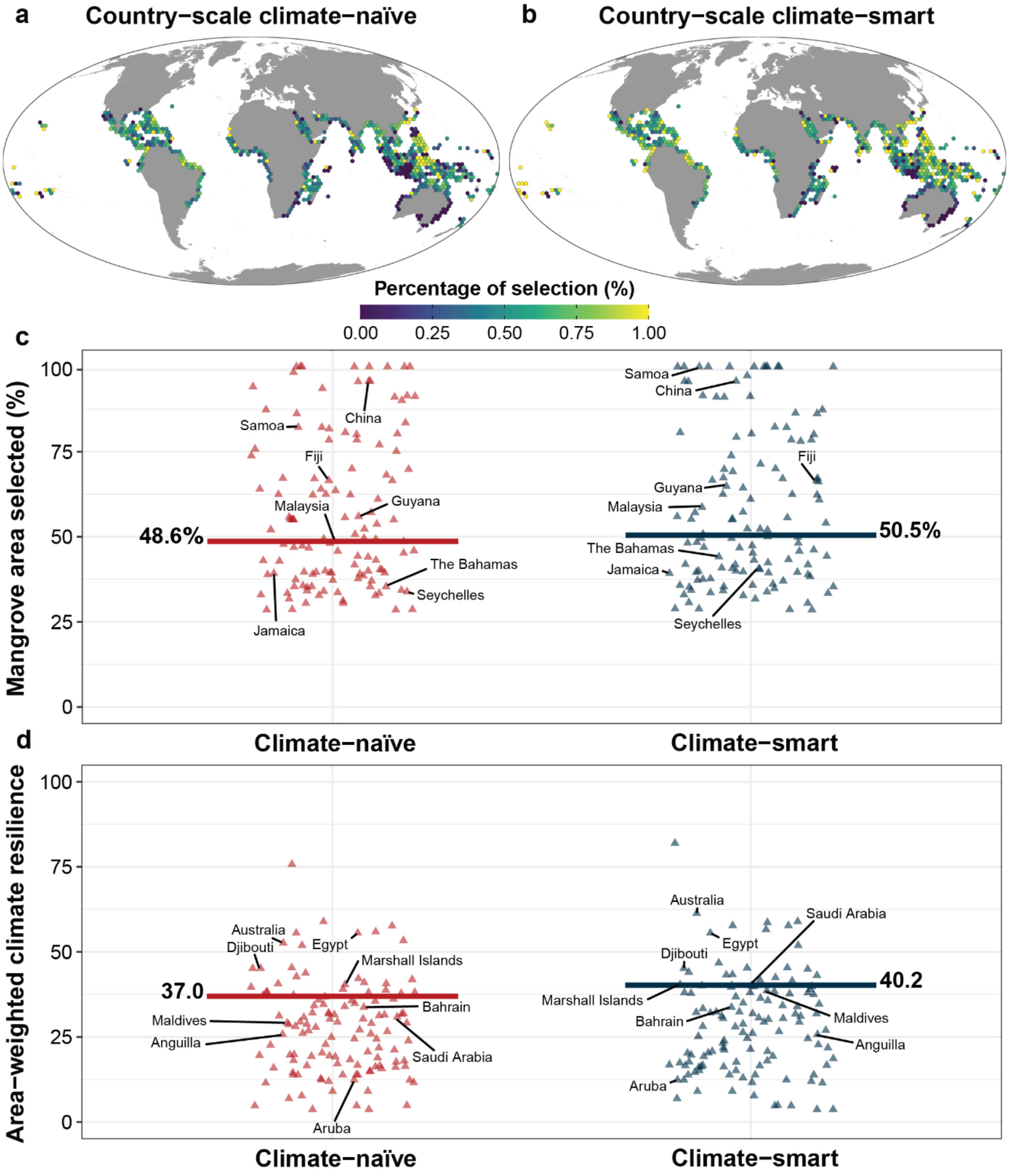

Supp. Fig. 1 | Country-scale results of the climate-naïve and climate-smart prioritisations. Results of the **a**-**b**, country-scale **a**, climate-naïve and **b**, climate-smart spatial prioritisation. Note that the % selection is shown because the original 631 km^2 for each planning unit in the analysis was aggregated at a resolution of ~63,000 km^2 for visualisation. **c**, % mangrove area selected and **d**, area-weighted climate resilience of the mangrove areas selected in the prioritisations by

country (each country is a triangle). Both prioritisations used a climate-smart threshold of 0.3. The horizontal lines show **c**, the total % of mangrove area selected and **d**, the global average climate resilience.

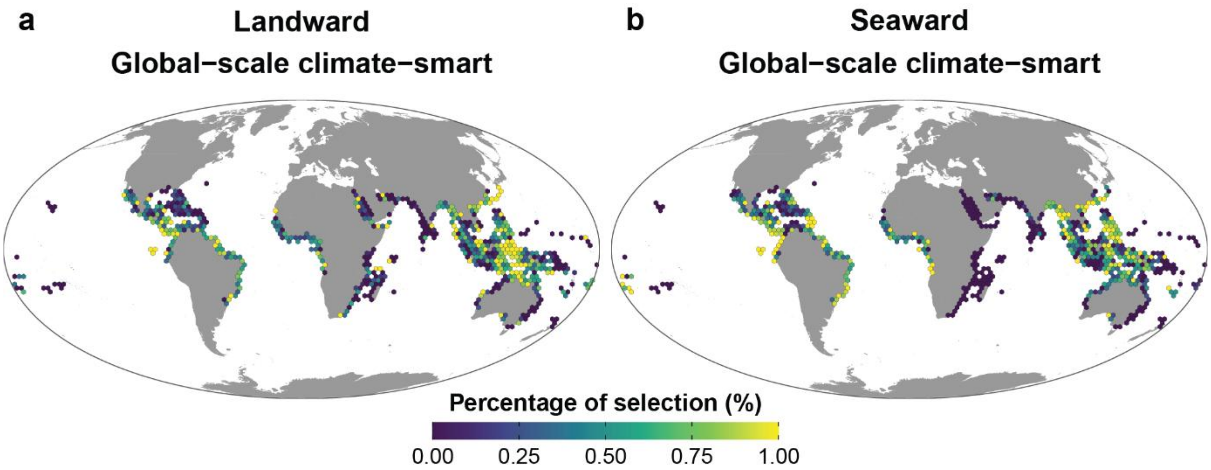

Supp. Fig. 2 | Global-scale climate-smart results of the landward and seaward prioritisations. Results of the **a-b**, global-scale climate smart **a**, landward and **b**, seaward spatial prioritisation. Note that the % selection is shown because the original 631 km^2 for each planning unit in the analysis was aggregated at a resolution of ~63,000 km^2 for visualisation.

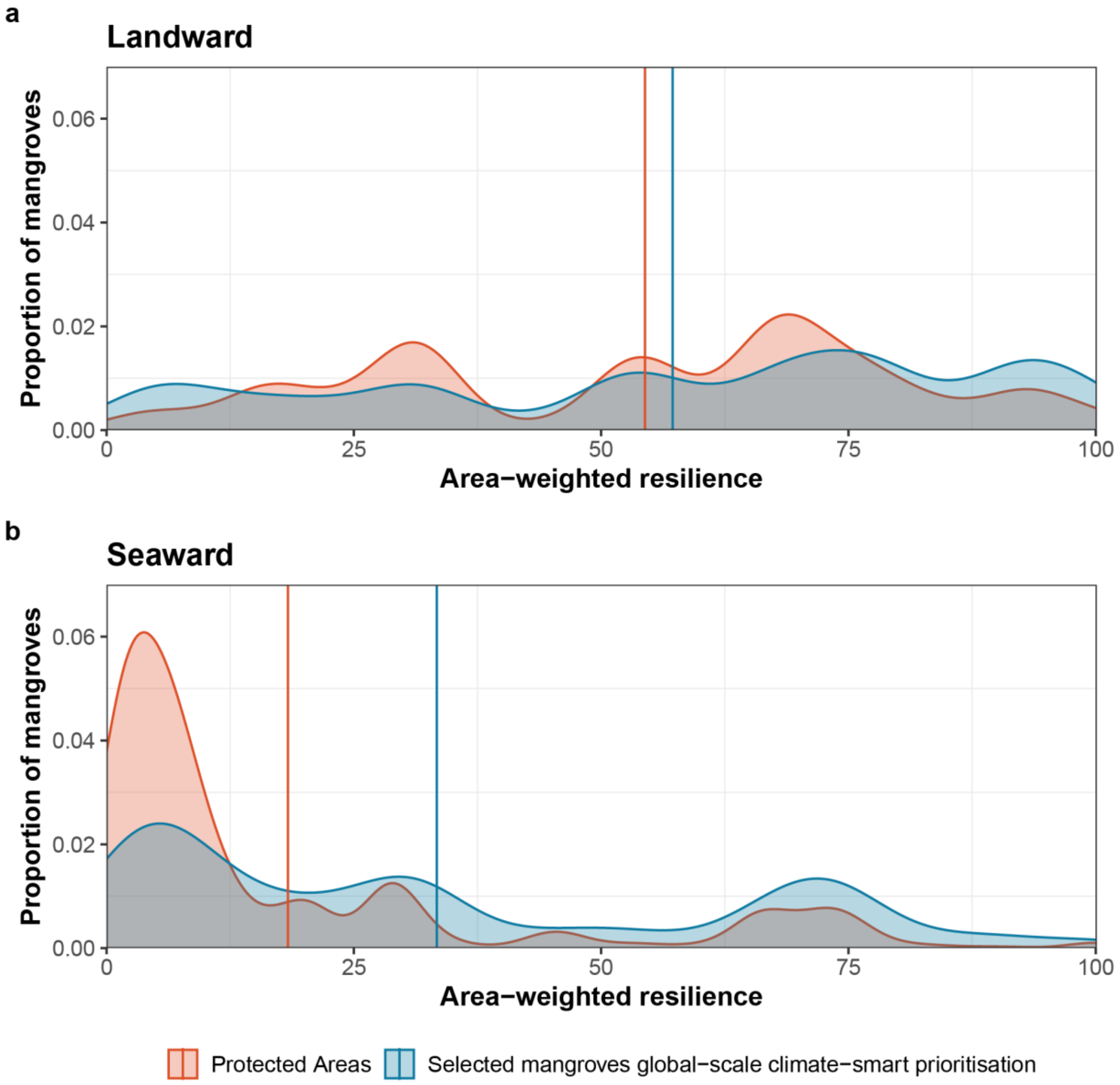

Supp. Fig. 3 | Protected area and associated resilience of landward and seaward networks. Kernel density plots reporting the area-weighted climate resilience of mangroves covered by protected areas, compared with those selected by our (a) landward and (b) seaward global-scale climate-smart prioritisations.

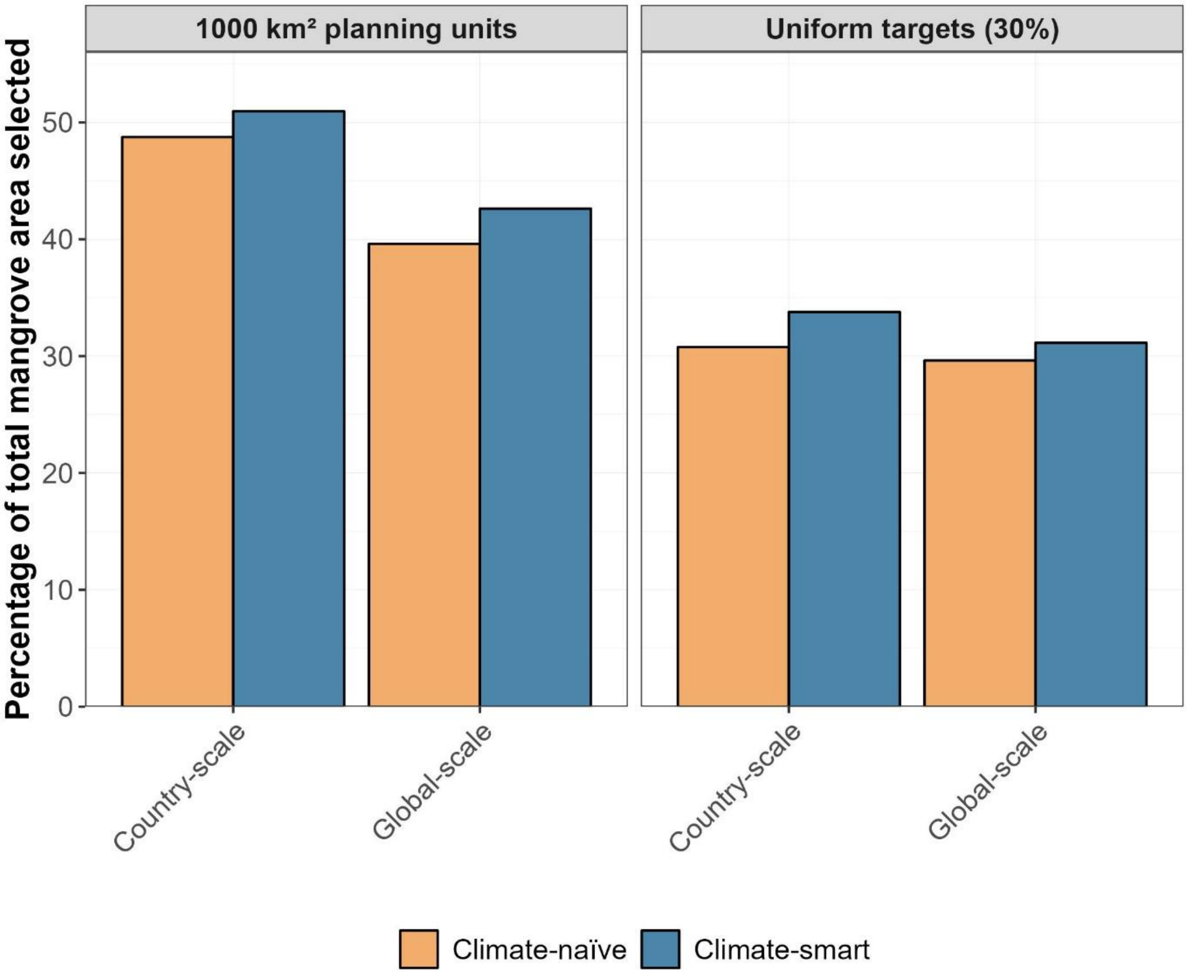

Supp. Fig. 4 | Percentage of total mangrove area selected by the prioritisations used for sensitivity analysis. Bar plots reporting the percentage of mangrove area selected by the climate-smart and climate-naïve, country-scale and global-scale prioritisations used for sensitivity analysis. These prioritisations used larger planning units (1000 km^2) than those of the baseline prioritisations (631 km^2) or uniform 30% conservation targets for each mangrove geomorphic species.

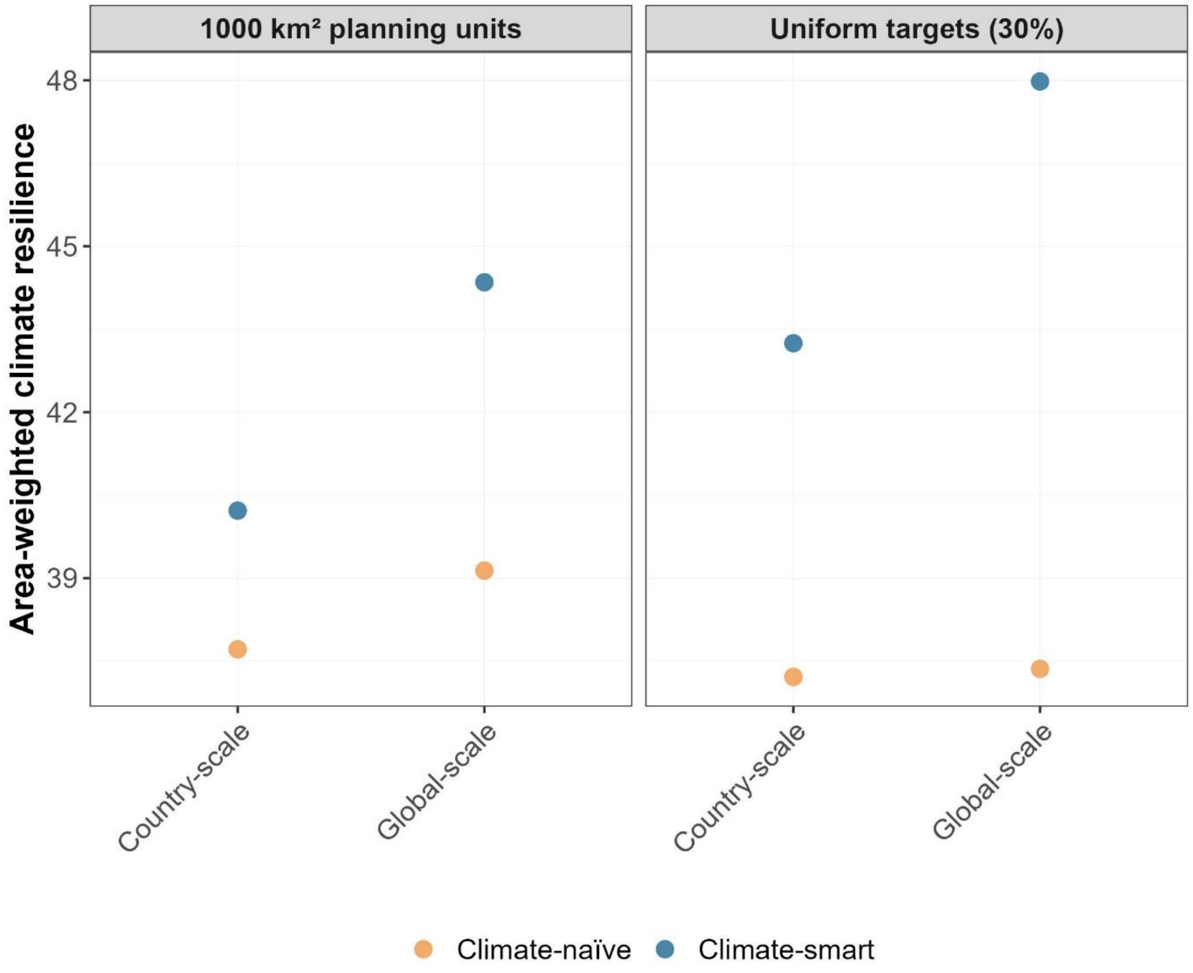

Supp. Fig. 5 | Average area-weighted climate resilience of mangroves selected by the prioritisations used for sensitivity analysis. Plots reporting the average climate resilience of mangrove area selected by the climate-smart and climate-naive, country-scale and global-scale prioritisations produced for the sensitivity analysis. These prioritisations used larger planning units (1000 km^2) than those of the baseline prioritisations (631 km^2) or uniform 30% conservation targets for each mangrove geomorphic species.

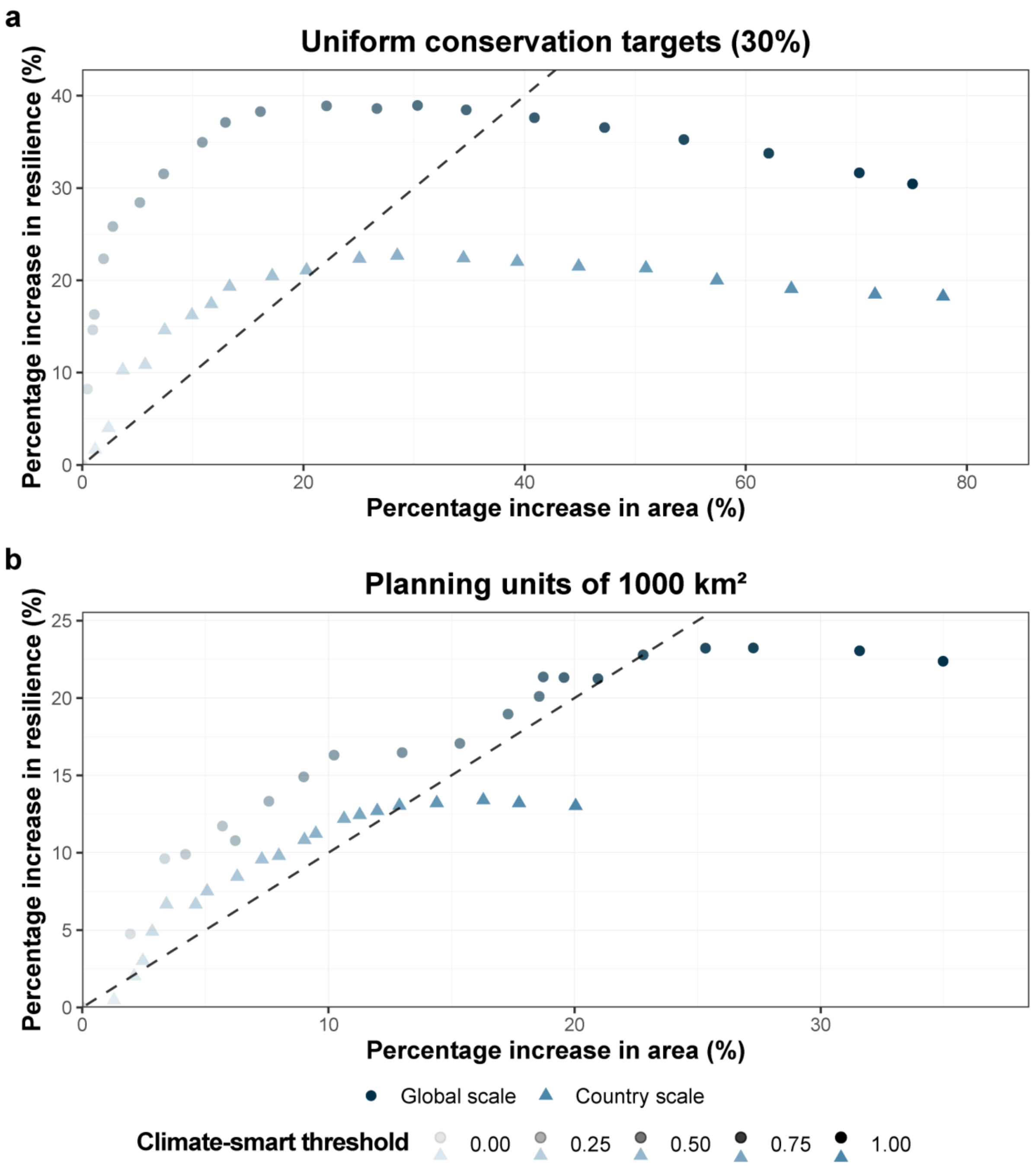

Supp. Fig. 6 | Sensitivity analysis of the differences in effectiveness of global-scale vs country-scale climate-smart spatial prioritisation. Percentage increase in area and resilience of the global-scale climate-smart prioritisation and of the country-scale climate-smart prioritisation from the respective baseline climate-naïve prioritisations, increasing climate-smart thresholds (0.05–1 with 0.05 increases—see Methods). These prioritisations used **a**, uniform 30% conservation targets for each geomorphic species, or **b**, larger planning units (1000 km^2) than those of the baseline prioritisations (631 km^2). The increase in climate-smart thresholds is displayed by an increase in the opacity of the dots and triangles. We added a 1:1 dashed line to aid interpretation.

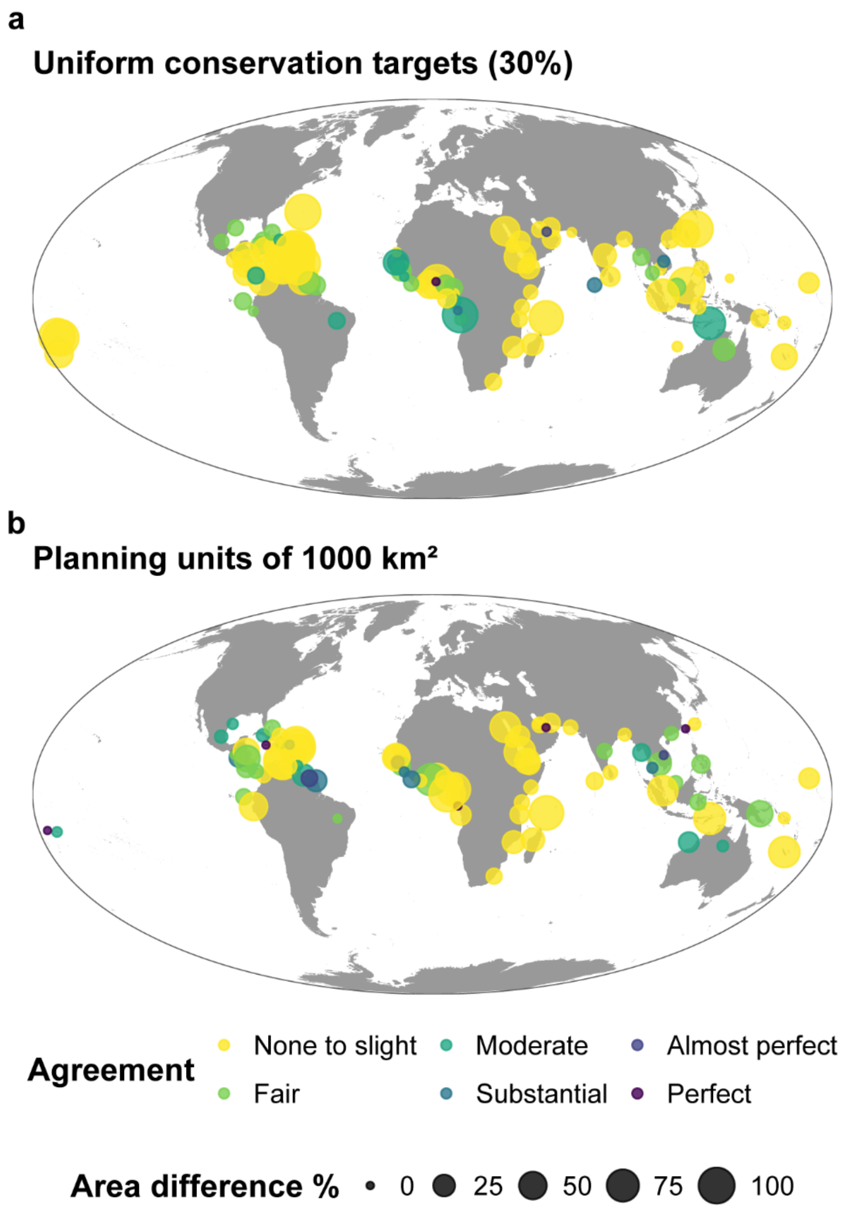

Supp. Fig. 7 | Sensitivity analysis of the differences between global-scale landward and seaward prioritisations by country. Each circle represents a country and is positioned in the centroid of the mangrove distribution in each country polygon. Circle colours represent the degree of agreement between the landward and seaward prioritisation calculated using Cohen's kappa statistic. Circle size represents the difference in the percentage of mangrove area selected between the two prioritisations (i.e., larger circles represent greater difference in total mangrove area selected in the country). These prioritisations used **a**, uniform 30% conservation targets for each geomorphic species, or **b**, larger planning units (1000 km^2) than those of the baseline prioritisations. Countries in which mangroves were not selected in both prioritisations are not represented. Both landward and seaward prioritisations used a climate-smart threshold of 0.3.

Supplementary Note 1 - List of the mangrove plant species included in the analysis.

Acanthus ebracteatus

Acanthus volubilis

Acrostichum aureum

Acrostichum danaeifolium

Acrostichum speciosum

Aegialitis annulata

Aegialitis rotundifolia

Aegiceras corniculatum

Aegiceras floridum

Aglaia cucullata

Avicennia alba

Avicennia bicolor

Avicennia germinans

Avicennia integra

Avicennia marina

Avicennia officinalis

Avicennia schaueriana

Brownlowia argentata

Brownlowia tersa

Bruguiera cylindrica

Bruguiera exaristata

Bruguiera gymnorrhiza

Bruguiera hainesii

Bruguiera parviflora

Bruguiera sexangula

Camptostemon philippinense

Camptostemon schultzii

Ceriops australis

Ceriops decandra

Ceriops tagal

Ceriops zippeliana

Conocarpus erectus

Dolichandrone spathacea

Excoecaria agallocha

Excoecaria indica

Heritiera fomes

Heritiera globosa

Heritiera littoralis

Kandelia candel

Kandelia obovata

Laguncularia racemosa

Lumnitzera littorea

Lumnitzera racemosa

Mora oleifera

Nypa fruticans

Osbornia octodonta

Pelliciera rhizophorae

Pemphis acidula

Phoenix paludosa

Rhizophora apiculata

Rhizophora mangle

Rhizophora mucronata

Rhizophora racemosa

Rhizophora samoensis

Rhizophora stylosa

Scyphiphora hydrophylacea

Sonneratia alba

Sonneratia apetala

Sonneratia caseolaris

Sonneratia griffithii

Sonneratia lanceolata

Sonneratia ovata

Tabebuia palustris

Xylocarpus granatum

Xylocarpus moluccensis
