# Supplementary material for: Safeguarding climate-resilient mangroves requires only a moderate increase in the global protected area
Source: Nat Commun. 2026 Jan 27;17:2063. doi: 10.1038/s41467-026-68877-4 (PMC12949150; doi:10.1038/s41467-026-68877-4)
Supplement: Supplementary file 3 — Reporting Summary [file 41467_2026_68877_MOESM3_ESM.pdf]

nature portfolio

Corresponding author(s): Dabalà Alvise

Last updated by author(s): 15/01/25

Reporting Summary

Nature Portfolio wishes to improve the reproducibility of the work that we publish. This form provides structure for consistency and transparency in reporting. For further information on Nature Portfolio policies, see our Editorial Policies and the Editorial Policy Checklist.

Statistics

For all statistical analyses, confirm that the following items are present in the figure legend, table legend, main text, or Methods section.

n/a	Confirmed	
☐	☒	The exact sample size (*n*) for each experimental group/condition, given as a discrete number and unit of measurement
☒	☐	A statement on whether measurements were taken from distinct samples or whether the same sample was measured repeatedly
☒	☐	The statistical test(s) used AND whether they are one- or two-sided<br>*Only common tests should be described solely by name; describe more complex techniques in the Methods section.*
☒	☐	A description of all covariates tested
☒	☐	A description of any assumptions or corrections, such as tests of normality and adjustment for multiple comparisons
☐	☒	A full description of the statistical parameters including central tendency (e.g. means) or other basic estimates (e.g. regression coefficient) AND variation (e.g. standard deviation) or associated estimates of uncertainty (e.g. confidence intervals)
☒	☐	For null hypothesis testing, the test statistic (e.g. *F*, *t*, *r*) with confidence intervals, effect sizes, degrees of freedom and *P* value noted<br>*Give P values as exact values whenever suitable.*
☒	☐	For Bayesian analysis, information on the choice of priors and Markov chain Monte Carlo settings
☒	☐	For hierarchical and complex designs, identification of the appropriate level for tests and full reporting of outcomes
☒	☐	Estimates of effect sizes (e.g. Cohen's *d*, Pearson's *r*), indicating how they were calculated

Our web collection on statistics for biologists contains articles on many of the points above.

Software and code

Policy information about availability of computer code

Data collection	We used data publicly available.
Data analysis	All analysis was conducted in R statistical computing environment (version 4.4.0). All prioritisations were completed using the prioritizr R package and Gurobi (v11.0.3; https://www.gurobi.com). Code to run the analysis in this study is currently available at: https://github.com/AlviDab/Mangroves_ClimateChange. Pending acceptance, code will be archived in a Zenodo digital repository.

For manuscripts utilizing custom algorithms or software that are central to the research but not yet described in published literature, software must be made available to editors and reviewers. We strongly encourage code deposition in a community repository (e.g. GitHub). See the Nature Portfolio guidelines for submitting code & software for further information.

Data

Policy information about availability of data

All manuscripts must include a data availability statement. This statement should provide the following information, where applicable:

- Accession codes, unique identifiers, or web links for publicly available datasets
- A description of any restrictions on data availability
- For clinical datasets or third party data, please ensure that the statement adheres to our policy

The datasets used in our study are publicly available online. These include: (1) the global map of mangroves is available for download from the Global Mangrove Watch website (https://www.globalmangrovewatch.org/); (2) IUCN distribution of mangrove species is available at: https://www.iucnredlist.org/resources/spatial-data-download; (3) the global biophysical mangrove typology is available for download from a Zenodo repository (https://zenodo.org/records/8340259); and (4) the

data on the probability of future mangrove loss (i.e., mangrove climate resilience) is available on Github: (https://github.com/cabuelow/mangrove-network-models?tab=readme-ov-file#probabilistic-forecasts-of-the-direction-of-future-change-in-mangrove-extent-using-network-models)
The datasets generated during the current study are currently available at: https://github.com/AlviDab/Mangroves_ClimateChange. Pending acceptance, these datasets will be archived in a Zenodo digital repository.

Research involving human participants, their data, or biological material

Policy information about studies with human participants or human data. See also policy information about sex, gender (identity/presentation), and sexual orientation and race, ethnicity and racism.

Reporting on sex and gender	This study does not include any human reseach participants.
Reporting on race, ethnicity, or other socially relevant groupings	This study does not include any human reseach participants.
Population characteristics	This study does not include any human reseach participants.
Recruitment	This study does not include any human reseach participants.
Ethics oversight	This study does not include any human reseach participants.

Note that full information on the approval of the study protocol must also be provided in the manuscript.

Field-specific reporting

Please select the one below that is the best fit for your research. If you are not sure, read the appropriate sections before making your selection.

☐ Life sciences ☐ Behavioural & social sciences ☒ Ecological, evolutionary & environmental sciences

For a reference copy of the document with all sections, see nature.com/documents/nr-reporting-summary-flat.pdf

Ecological, evolutionary & environmental sciences study design

All studies must disclose on these points even when the disclosure is negative.

Study description	We evaluated the efficiency of protecting mangroves in climate-smart versus climate-naïve reserve networks.
Research sample	We obtained a fine-scale global distribution map of mangroves using the Global Mangrove Watch (GMW). We obtained geographic range data of the 65 most common mangrove species from the IUCN Red List of Threatened Species. Mangrove biophysical typology data were included in the analysis to define a system that ensures the greatest benefits for biodiversity representation. We also obtained data on the probability of future mangrove loss (i.e., mangrove climate resilience) that is available on Github: (https://github.com/cabuelow/mangrove-network-models?tab=readme-ov-file#probabilistic-forecasts-of-the-direction-of-future-change-in-mangrove-extent-using-network-models). Protected area data were obtained from the World Database on Protected Areas (WDPA).
Sampling strategy	We subdivided the study area into 7,559 hexagonal planning units, each with a spatial resolution of 631 km^2 (~0.25° alongshore).
Data collection	We used data publicly available.
Timing and spatial scale	We used global mangrove distribution data from 2022. Probability of future mangrove loss used mid-term projections (years 2040-2060).
Data exclusions	No data were excluded from the analysis.
Reproducibility	Code to run the analysis in this study is currently available at: https://github.com/AlviDab/Mangroves_ClimateChange. The code is archived in a Zenodo digital repository: https://zenodo.org/records/17728764.
Randomization	This study does not include any level of randomization.
Blinding	Data were not blinded in this analysis.

Did the study involve field work? ☐ Yes ☒ No

Reporting for specific materials, systems and methods

We require information from authors about some types of materials, experimental systems and methods used in many studies. Here, indicate whether each material, system or method listed is relevant to your study. If you are not sure if a list item applies to your research, read the appropriate section before selecting a response.

Materials & experimental systems

n/a	Involved in the study
☒	☐ Antibodies
☒	☐ Eukaryotic cell lines
☒	☐ Palaeontology and archaeology
☒	☐ Animals and other organisms
☒	☐ Clinical data
☒	☐ Dual use research of concern
☒	☐ Plants

Methods

n/a	Involved in the study
☒	☐ ChIP-seq
☒	☐ Flow cytometry
☒	☐ MRI-based neuroimaging

Plants

Seed stocks	*Report on the source of all seed stocks or other plant material used. If applicable, state the seed stock centre and catalogue number. If plant specimens were collected from the field, describe the collection location, date and sampling procedures.*
Novel plant genotypes	*Describe the methods by which all novel plant genotypes were produced. This includes those generated by transgenic approaches, gene editing, chemical/radiation-based mutagenesis and hybridization. For transgenic lines, describe the transformation method, the number of independent lines analyzed and the generation upon which experiments were performed. For gene-edited lines, describe the editor used, the endogenous sequence targeted for editing, the targeting guide RNA sequence (if applicable) and how the editor was applied.*
Authentication	*Describe any authentication procedures for each seed stock used or novel genotype generated. Describe any experiments used to assess the effect of a mutation and, where applicable, how potential secondary effects (e.g. second site T-DNA insertions, mosiacism, off-target gene editing) were examined.*
